# [Tpy_2_Co][Co(CO)_4_]: a mixed-valent cobalt(III)/cobalt(−I) com­plex based on phenyl­tris­(pyridin-2-yl)borate (Tpy^−^)

**DOI:** 10.1107/S2056989026004093

**Published:** 2026-05-22

**Authors:** Oshani Wijesinghe, Robert Joseph Comito

**Affiliations:** a The University of Houston, 4800 Calhoun Road Houston TX 77004 USA; Texas A & M University, USA

**Keywords:** crystal structure, scorpionate, tris­(pyridin-2-yl)borate, mixed valence

## Abstract

Tris(pyridin-2-yl)borates are an emerging class of scorpionate ligands. Distinguished by their robustness and nucleophilicity, they have promise as alternatives to tris­(pyrazol­yl)borates in coordination chemistry, materials ap­plications, and catalysis. In this article, bis­[phenyl­tris­(pyridin-2-yl)borato]cobalt(III) tetra­carbonyl­cobalt(–I) di­chloro­methane monosolvate, [Co(C_21_H_17_BN_3_)_2_][Co(CO)_4_]·CH_2_Cl_2_ or [Tpy_2_Co][Co(CO)_4_]·CH_2_Cl_2_, was prepared by the reaction of hy­dro­gen phenyl­tris­(pyridin-2-yl)borate (TpyH), tri­ethyl­amine, and Co(CO)_4_I. Single-crystal X-ray diffraction resulted in a homoleptic com­plex consisting of the diamagnetic [Tpy_2_Co]^+^ cation and the tetra­car­bonyl­cobaltate {[Co(CO)_4_]^−^} counter-ion. The unusual Co^III^/Co^—I^ mixed-valence com­position presumably results from disproportionation. [Tpy_2_Co][Co(CO)_4_] represents the first mixed-valence tris­(pyridin-2-yl)borate com­plex.

## Abstract 2

The reaction of hy­dro­gen phenyl­tris­(pyridin-2-yl)borate (TpyH), tri­ethyl­amine, and Co(CO)_4_I gives a mixed-valent salt [Tpy_2_Co][Co(CO)_4_], consisting of a homoleptic cobalt(III) com­plex and a cobalt(–I) counter-ion. [Tpy_2_Co][Co(CO)_4_] represents the first mixed-valent tris­(pyridin-2-yl)borate com­plex.

## 1. Chemical context

Scorpionate ligands have a wide range of applications in inorganic chemistry and are typified by the tris­(pyrazol­yl)borate ligands introduced by Trofimenko (Calabrese *et al.*, 1986 ▸; Trofimenko, 1966 ▸; Trofimenko, 1967 ▸; Trofimenko, 1993 ▸; Santini *et al.*, 2010 ▸). A notable weakness of tris­(pyrazol­yl)borates is the vulnerability of their metal com­plexes to hydrolysis and borotropic shifts (Albinati *et al.*, 1997 ▸; Biagini *et al.*, 2006 ▸; Chisholm *et al.*, 1996 ▸; Darensbourg *et al.*, 1996 ▸; Kunrath *et al.*, 2003 ▸; Lee & Jordan, 2005 ▸; Michiue & Jordan, 2004 ▸; Trofimenko *et al.*, 1989 ▸). In this context, aryl­tris­(pyridin-2-yl)borates have emerged as more robust and electron-donating alternatives (McQuade & Jäkle, 2023 ▸; Pawar *et al.*, 2016 ▸), which lack the labile B—N and B—H bonds of tris­(pyrazol­yl)borates and offer advantages for catalysis or coordination studies under demanding conditions. Analogous to tris­(pyrazol­yl)borates, aryl­tris­(pyridin-2-yl)borates are anionic tripodal ligands with a tendency for facial κ^3^-coordination geometries. Also like tris­(pyrazol­yl)borates (Abernethy *et al.*, 2008 ▸; O’Reilly *et al.*, 1996 ▸), aryl­tris­(pyridin-2-yl)borates show a strong preference for octa­hedral coordination geometries in metal com­plexes, often as 2:1 homoleptic com­plexes. Jäkle introduced (Cui *et al.*, 2012 ▸) and subsequently studied (Cui *et al.*, 2013 ▸; Goura *et al.*, 2022 ▸; Jeong *et al.*, 2016 ▸; Pawar *et al.*, 2015 ▸; Shipman *et al.*, 2013 ▸) the aryl­tris­(pyridin-2-yl)borates as octa­hedral 2:1 homoleptic com­plexes of divalent and trivalent metal ions (Mg^2+^, Mn^2+^, Fe^2+^, Fe^3+^, Cu^2+^, and Ru^2+^). Our laboratory reported 1:1 com­plexes of phenyl­tris­(pyridin-2-yl)borate (Tpy^−^) of V^5+^ and V^3+^ that are also octa­hedral·(Qian & Comito, 2021 ▸; Qian & Comito, 2023 ▸). We did obtain tetra­hedral organozinc, organoaluminum, and organogallium com­plexes by reaction of TpyH (**1**) with di­ethyl­zinc, with tri­alkyl­aluminums, and with tri­methyl­gallium (Qian *et al.*, 2024 ▸). However, the reaction of TpyH (**1**) with metal amides *M*(HMDS)_2_; *M*^2+^ = Mg^2+^, Zn^2+^, or Ca^2+^; HMDS^−^ = ^−^N(SiMe_3_)_2_] instead gave homoleptic com­plexes Tpy_2_M (Qian & Comito, 2022 ▸).

**Figure 1:**
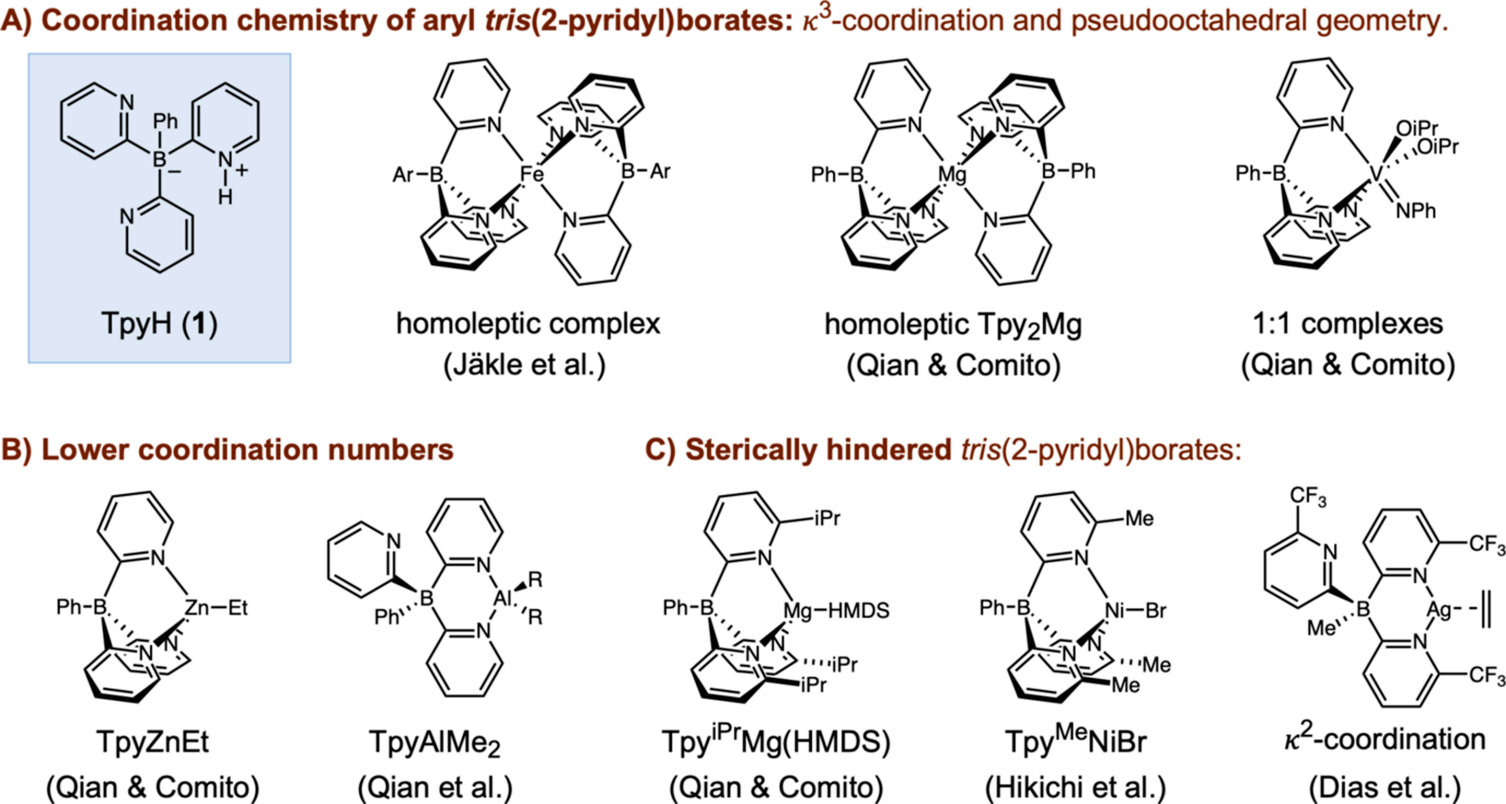
Published aryl­tris­(pyridin-2-yl)borates: (*a*) octa­hedral tris­(pyridin-2-yl)borate metal com­plexes, (*b*) tetra­hedral organometallic tris­(pyridin-2-yl)borate com­plexes and (*c*) sterically hindered tris­(pyridin-2-yl)borates with lower coordination numbers.

Consequently, all other metal tris­(pyridin-2-yl)borate com­plexes with coordination numbers less than 6 have used ligands with sterically hindered six-substituted pyridin-2-yl units, which sterically inhibit homoleptic com­plex formation. Our group introduced the hy­dro­gen phenyl­tris­(6-*R*-pyridin-2-yl)borate Tpy^*R*^H proligands (*R*^−^ = mesityl, *tert*-butyl, and isoprop­yl). From them we prepared the four-coordinate and dis­torted tetra­hedral com­plexes Tpy^iPr^Mg(HMDS), Tpy^iPr^Zn(HMDS), Tpy^iPr^Ca(HMDS), and Tpy^tBu^Ca(HMDS) (Qian & Comito, 2022 ▸). Hikichi reported the more ideally tetra­hedral com­plex Tpy^Me^NiBr, prepared from hy­dro­gen phenyl­tris­(6-methyl­pyridin-2-yl)borate (Tpy^Me^H) (Fujiwara *et al.* 2022 ▸). Dias studied low-coordinate tris­(6-tri­fluoro­methyl­pyridin-2-yl)borate com­plexes of coinage metals (Vanga *et al.*, 2022 ▸; Watson *et al.*, 2023 ▸; Vanga *et al.*, 2024 ▸) and of thallium (Vanga *et al.*, 2023 ▸), com­paring and contrasting them to tri­fluoro­methyl­ated tris­(pyrazol­yl)borate com­plexes that they also reported (Dias *et al.*, 1996 ▸; Dias & Kim, 1996 ▸; Dias & Lovely, 2008 ▸; Dias & Jin, 2003 ▸) (Fig. 1 ▸).

**Figure 2:**
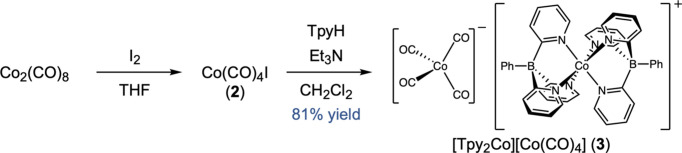
The synthesis of the title com­pound (**3**).

In this context, we targeted a five-coordinate com­plex TpyCo(CO)_2_ as a platform for coordination chemistry and catalysis. Transition-metal carbonyl com­plexes of Tpy^−^ would be analogs of the well-known ‘piano-stool’ com­plexes of cyclo­penta­dienides (Poli, 1990 ▸; Kuo *et al.*, 2018 ▸), which often have coordination numbers higher or lower than 6. Given the structural analogy to CpCo(CO)_2_ (Nafady *et al.*, 2006 ▸) and given that TpyCo(CO)_2_ would be 18-electron at cobalt, we reasoned that this com­plex would be stable despite the lower coordination number. However, the reaction of TpyH (**1**) and tri­ethyl­amine with Co(CO)_4_I (**2**), prepared freshly from Co_2_(CO)_8_ and I_2_, instead gave com­plex [Tpy_2_Co][Co(CO)_4_] (**3**) in 81% yield (Fig. 2 ▸). ^1^H and ^13^C NMR analysis of this com­plex results in diamagnetic signals for the Tpy^−^ unit, indicating a low-spin *d*^6^ [Tpy_2_Co]^+^ cation. IR spectroscopy identifies a ν(C=O) stretch at 1980 cm^−1^, consistent with the Co(CO)_4_^−^ anion (Brennessel & Ellis, 2014 ▸). That would make [Tpy_2_Co][Co(CO)_4_] (**3**) the first mixed-valent tris­(pyridin-2-yl)borate com­plex. The Co^III^/Co^–I^ mixed valence is rare, although mixed-valent cobalt com­plexes with a Co(CO)_4_^−^ anion are known (see *Database survey*) (Fig. 2 ▸).

The structures we obtained reinforce the notion of tris­(pyridin-2-yl)borates as ‘octa­hedral enforcers’, which like tris­(pyrazol­yl)borates show a much stronger preference for octa­hedral coordination geometries than do cyclo­penta­dienides.

## 2. Structural commentary

**Figure 3:**
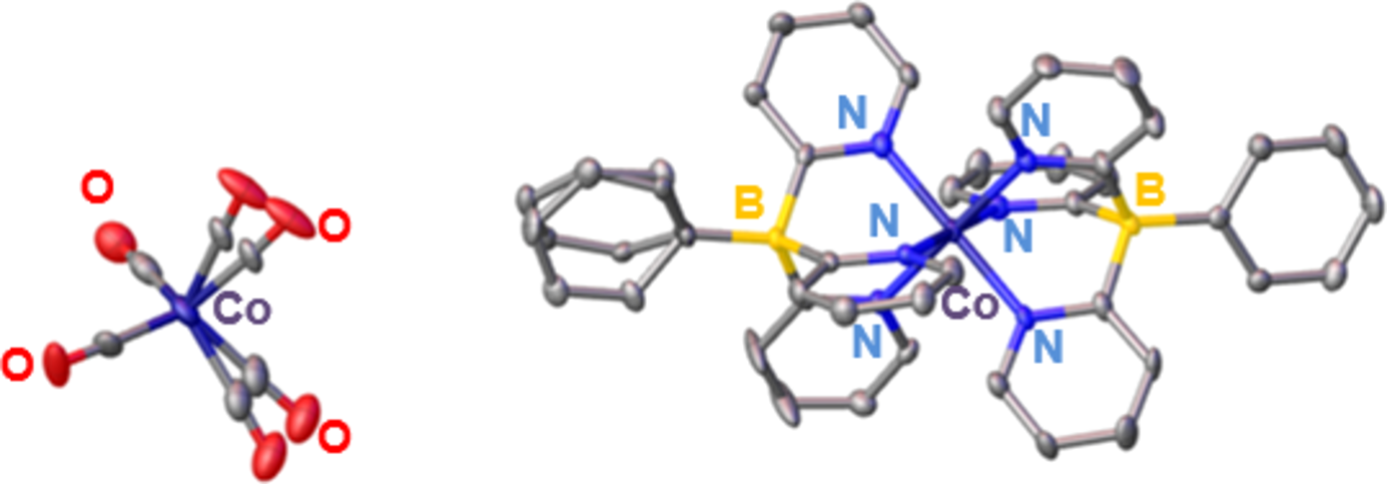
SCXRD structure of [Tpy_2_Co][Co(CO)_4_] (**3**), with displacement ellipsoids drawn at the 50% probability level for all atoms except hy­dro­gen and the solvent (CH_2_Cl_2_). All atoms are labeled except for carbon.

The [Tpy_2_Co]^+^ com­plex showed nearly ideal octa­hedral coordination, with six N—Co bond lengths between 1.939 (6) and 1.979 (5) Å, and six intra­ligand N—Co—N bond angles between 90.4 (2) and 91.2 (2)°. The nearly linear N—Co—N angles of ∼179° confirms the symmetry of the com­plex (Fig. 3 ▸).

## 3. Supra­molecular features

There are no significant inter­molecular inter­actions between the [Tpy_2_Co]^+^ and [Co(CO)_4_]^−^ ions.

## 4. Database survey

A database survey found many single-crystal X-ray diffraction structures of mixed-valent Co^I^/Co^–I^ (Hollingsworth *et al.*, 2018 ▸; Adamczyk *et al.*, 2011 ▸; Azhakar *et al.*, 2012 ▸; Luque-Gómez *et al.*, 2023 ▸; Luque-Gómez *et al.*, 2025 ▸; Chen *et al.*, 2025 ▸) and Co^II^/Co^–I^ (Uehara *et al.*, 2005 ▸; Wang *et al.*, 2022 ▸; Kaefer *et al.*, 2021 ▸) com­plexes with a [Co(CO)_4_]^−^ ion (not an exhaustive list). Co^III^/Co^–I^ com­plexes with a [Co(CO)_4_]^−^ ion are rare (Petz *et al.*, 2006 ▸) with the closest analog being [Cp_2_Co][Co(CO)_4_] (Cp^−^ = cyclo­penta­dienide) (Bockman & Kochi, 1988 ▸). Inter­estingly the published [Cp_2_Co][Co(CO)_4_] salt is pale yellow like [Tpy_2_Co][Co(CO)_4_] (**3**) reported here.

## 5. Synthesis and crystallization

### 5.1. General comments

All manipulations were performed in a nitro­gen glovebox using dry degassed solvents unless otherwise noted. Hydrogen phenyl­tris­(pyridin-2-yl)borate (TpyH, **1**) was prepared ac­cording to our previous report (Qian & Comito, 2021 ▸). NMR spectra were recorded on a JEOL 400 MHz spectrometer. ^1^H NMR signals are inter­nally referenced relative to residual proton solvent signals (chloro­form-*d* at δ = 7.26 ppm). ^13^C NMR signals are inter­nally referenced relative to the solvent signal (chloro­form-*d* at δ = 77.16). Data for ^1^H NMR are reported as follows: chemical shift (δ, ppm), multiplicity (*s* = singlet, *d* = doublet), integration, and coupling constant (Hz). Data for ^13^C NMR are reported in terms of chemical shift and multiplicity where appropriate. IR spectra were recorded on a Bruker Platinum ATR spectrometer with monolithic diamond crystal plate and are reported in terms of wavenumber of absorption (cm^−1^). X-ray diffraction data were collected on a Bruker DUO platform diffractometer equipped with a 4K CCD APEXII detector and an Incoatec 30 W Cu microsource with com­pact multilayer optics. Data were collected using a narrow-frame algorithm with scan widths of 0.50° in ω and a θ dependent exposure time of 10–30 s/frame at 4 cm detector distance.

### 5.2. Bis[phenyl­tris­(pyridin-2-yl)borato]cobalt(II) tetra­car­bon­yl­cobalt(−I), [Tpy_2_Co][Co(CO)_4_] (3)

A solution of I_2_ (10 mg, 0.039 mmol, 0.66 equiv.) and tetra­hydro­furan (2.0 ml) was added to a solution of Co_2_(CO)_8_ (11 mg, 0.032 mmol, 0.54 equiv.) and tetra­hydro­furan (2.0 ml). The combination resulted in a green color. After 2  h in the dark, the solution was concentrated under vacuum to a green solid. The solid was redissolved in CH_2_Cl_2_ (2.0 ml) and transferred to a solution of hy­dro­gen phenyl­tris­(pyridin-2-yl)borate (TpyH, **1**; 19.4 mg, 0.061 mmol, 1.0 equiv.), tri­ethyl­amine (9.0 µl, 0.065 mmol, 1.1 equiv.), and CH_2_Cl_2_ (2.0 ml). After stirring for 18 h in the dark, the reaction was concentrated under vacuum, giving a bright-yellow solid. Crude ^1^H NMR analysis resulted in a mixture of the isolated product and a tri­ethyl­amine-derived product, presumably tri­ethyl­ammonium iodide. The crude product was then removed from the glovebox and partitioned between CH_2_Cl_2_ (10 ml) and H_2_O (10 ml) in a separatory funnel. The aqueous phase was then extracted with CH_2_Cl_2_ (3 × 10 ml). The combined CH_2_Cl_2_ solution was dried over anhydrous Na_2_SO_4_ and then filtered. Vacuum drying yielded the title com­pound as a dark-green powder (yield: 0.073 g, 0.081 mmol, 81%). ^1^H NMR (400 MHz, CDCl_3_): δ 8.08 (*s*, 4H), 7.88 (*s*, 6H), 7.59 (*d*, *J* = 54.6 Hz, 12H), 6.79 (*d*, *J* = 49.5 Hz, 12H). These NMR data matched those of the crude product, before exposure to air and water. ^13^C NMR (101 MHz, CDCl_3_) δ 154.3, 137.5, 136.0, 129.7, 128.5, 126.4, 123.6. The two *ipso*-C atoms (B—**C**) were not observed. IR 2934, 2761, 2679, 2477, 1980, 1900, 1593, 1459, 1419, 1272, 1217, 1163, 1069, 1031, 887, 765, 739, 713, 639, 548, 468 cm^−1^. Elemental analysis for C_40_H_24_B_2_N_6_O_4_Co_2_, calculated (%): C 60.55, H 3.05, N 10.61; found (%): C 59.66, H 3.5, N 9.52; difference (%) C 0.89, H 0.45, N 1.09. A crystal suitable for single-crystal X-ray diffraction was obtained as a green needle by vapor diffusion of hexane into a di­chloro­methane solution of **3** at room temperature in the glovebox.

## 6. Refinement

All of the H atoms were calculated in idealized positions and refined riding on their parent atoms. Crystal data, data collection and structure refinement details are summarized in Table 1 ▸.

## Supplementary Material

Crystal structure: contains datablock(s) I. DOI: 10.1107/S2056989026004093/jy2070sup1.cif

Structure factors: contains datablock(s) I. DOI: 10.1107/S2056989026004093/jy2070Isup2.hkl

Spectroscopic data for compound 3. DOI: 10.1107/S2056989026004093/jy2070sup3.pdf

CCDC reference: 2547200

Additional supporting information:  crystallographic information; 3D view; checkCIF report

## Figures and Tables

**Table 1:** Experimental details

Crystal data	
Chemical formula	[Co(C_21_H_17_BN_3_)_2_][Co(CO)_4_]·CH_2_Cl_2_
*M*_r_	959.20
Crystal system, space group	Monoclinic, *Cc*
Temperature (K)	100
*a*, *b*, *c* (Å)	9.2341 (5), 22.3721 (13), 20.8218 (14)
β (°)	97.593 (3)
*V* (Å^3^)	4263.8 (4)
*Z*	4
Radiation type	Mo *K*α
μ (mm^−1^)	0.96
Crystal size (mm)	0.27 × 0.25 × 0.01

Data collection	
Diffractometer	Bruker D8 Venture
Absorption correction	Empirical (using intensity measurements) (*SADABS*; Bruker, 2025 ▸)
*T*_min_, *T*_max_	0.643, 0.746
No. of measured, independent and observed [*I* > 2σ(*I*)] reflections	45755, 10229, 7481
*R*_int_	0.117
(sin θ/λ)_max_ (Å^−1^)	0.667

Refinement	
*R*[*F*^2^ > 2σ(*F*^2^)], *wR*(*F*^2^), *S*	0.061, 0.125, 1.01
No. of reflections	10229
No. of parameters	732
No. of restraints	293
H-atom treatment	H-atom parameters constrained
Δρ_max_, Δρ_min_ (e Å^−3^)	0.83, −0.51
Absolute structure	Flack *x* determined using 2647 quotients [(I+)-(I-)]/[(I+)+(I-)] (Parsons *et al.*, 2013 ▸)
Absolute structure parameter	−0.015 (13)

Computer programs: *APEX6* (Bruker, 2025 ▸), *SAINT* (Bruker, 2025 ▸), *SHELXT2018* (Sheldrick, 2015*a* ▸) and *SHELXL2025* (Sheldrick, 2015*b* ▸).

## supplementary crystallographic information

### Bis[phenyltris(pyridin-2-yl)borato]cobalt(III) tetracarbonylcobalt(-I) dichloromethane monosolvate Crystal data

**Table:**

[Co(C_21_H_17_BN_3_)_2_][Co(CO)_4_]·CH_2_Cl_2_	*F*(000) = 1960
*M**_r_* = 959.20	*D*_x_ = 1.494 Mg m^−^^3^
Monoclinic, *C**c*	Mo *K*α radiation, λ = 0.71073 Å
*a* = 9.2341 (5) Å	Cell parameters from 4368 reflections
*b* = 22.3721 (13) Å	θ = 2.4–20.0°
*c* = 20.8218 (14) Å	µ = 0.96 mm^−^^1^
β = 97.593 (3)°	*T* = 100 K
*V* = 4263.8 (4) Å^3^	Plate, brown
*Z* = 4	0.27 × 0.25 × 0.01 mm

### Bis[phenyltris(pyridin-2-yl)borato]cobalt(III) tetracarbonylcobalt(-I) dichloromethane monosolvate Data collection

**Table:**

Bruker D8 Venture diffractometer	7481 reflections with *I* > 2σ(*I*)
φ and ω scans	*R*_int_ = 0.117
Absorption correction: empirical (using intensity measurements) (SADABS; Bruker, 2025)	θ_max_ = 28.3°, θ_min_ = 2.1°
*T*_min_ = 0.643, *T*_max_ = 0.746	*h* = −12→12
45755 measured reflections	*k* = −29→29
10229 independent reflections	*l* = −27→26

### Bis[phenyltris(pyridin-2-yl)borato]cobalt(III) tetracarbonylcobalt(-I) dichloromethane monosolvate Refinement

**Table:**

Refinement on *F*^2^	Secondary atom site location: difference Fourier map
Least-squares matrix: full	Hydrogen site location: inferred from neighbouring sites
*R*[*F*^2^ > 2σ(*F*^2^)] = 0.061	H-atom parameters constrained
*wR*(*F*^2^) = 0.125	*w* = 1/[σ^2^(*F*_o_^2^) + (0.0305*P*)^2^ + 7.1252*P*] where *P* = (*F*_o_^2^ + 2*F*_c_^2^)/3
*S* = 1.01	(Δ/σ)_max_ < 0.001
10229 reflections	Δρ_max_ = 0.83 e Å^−^^3^
732 parameters	Δρ_min_ = −0.51 e Å^−^^3^
293 restraints	Absolute structure: Flack *x* determined using 2647 quotients [(I+)-(I-)]/[(I+)+(I-)] (Parsons *et al.*, 2013)
Primary atom site location: structure-invariant direct methods	Absolute structure parameter: −0.015 (13)

### Bis[phenyltris(pyridin-2-yl)borato]cobalt(III) tetracarbonylcobalt(-I) dichloromethane monosolvate Special details

**Table:**

Geometry. All esds (except the esd in the dihedral angle between two l.s. planes) are estimated using the full covariance matrix. The cell esds are taken into account individually in the estimation of esds in distances, angles and torsion angles; correlations between esds in cell parameters are only used when they are defined by crystal symmetry. An approximate (isotropic) treatment of cell esds is used for estimating esds involving l.s. planes.
Refinement. All measurements were made with a Bruker D8 Venture four-circle diffractometer equipped with a Photon III detector, Oxford Cryostream 1000 cooling device and Cu/Mo Incoatec microfocus IµS 3.0 sources with HELIOS mirror optics. The data were integrated using the Bruker SAINT program, with the intensities corrected for Lorentz factor, polarization, and background effects. The data were scaled, and an absorption correction was applied using SADABS. The structure was solved with the program SHELXT 2018, and refined with SHELXL 2025 using full-matrix least-squares refinement. The non-H atoms were refined with anisotropic thermal parameters, and all of the H atoms were calculated in idealized positions and refined riding on their parent atoms.

### Bis[phenyltris(pyridin-2-yl)borato]cobalt(III) tetracarbonylcobalt(-I) dichloromethane monosolvate Fractional atomic coordinates and isotropic or equivalent isotropic displacement parameters (Å^2^)

**Table:**

	*x*	*y*	*z*	*U*_iso_*/*U*_eq_	Occ. (<1)
Co1	0.16619 (10)	0.42442 (4)	0.39521 (6)	0.01777 (19)	
N1	0.0949 (6)	0.3467 (2)	0.4175 (3)	0.0207 (12)	
N2	−0.0339 (5)	0.4516 (2)	0.3639 (3)	0.0193 (12)	
N3	0.1904 (5)	0.3961 (2)	0.3078 (3)	0.0173 (12)	
N4	0.2414 (5)	0.5026 (2)	0.3731 (3)	0.0202 (12)	
N5	0.1423 (5)	0.4522 (2)	0.4814 (3)	0.0200 (12)	
N6	0.3672 (5)	0.3969 (2)	0.4258 (3)	0.0192 (12)	
C1	−0.1833 (6)	0.3069 (3)	0.2660 (3)	0.0177 (13)	
C2	−0.3318 (7)	0.3067 (3)	0.2743 (4)	0.0260 (16)	
H2	−0.365424	0.335361	0.302539	0.031*	
C3	−0.4314 (8)	0.2663 (3)	0.2432 (4)	0.0316 (17)	
H3	−0.531168	0.268373	0.249736	0.038*	
C4	−0.3867 (8)	0.2236 (3)	0.2029 (4)	0.0325 (18)	
H4	−0.455041	0.196443	0.180791	0.039*	
C5	−0.2410 (8)	0.2206 (3)	0.1951 (4)	0.0263 (16)	
H5	−0.208222	0.190819	0.167864	0.032*	
C6	−0.1422 (7)	0.2608 (3)	0.2266 (3)	0.0199 (14)	
H6	−0.041971	0.256948	0.221243	0.024*	
C7	−0.0093 (7)	0.3194 (3)	0.3766 (3)	0.0192 (14)	
C8	−0.0670 (8)	0.2648 (3)	0.3957 (4)	0.0294 (17)	
H8	−0.140322	0.244886	0.367158	0.035*	
C9	−0.0180 (9)	0.2399 (3)	0.4554 (4)	0.0328 (18)	
H9	−0.058591	0.203528	0.468251	0.039*	
C10	0.0908 (8)	0.2685 (3)	0.4962 (4)	0.0336 (18)	
H10	0.127383	0.251900	0.537098	0.040*	
C11	0.1444 (8)	0.3216 (3)	0.4759 (4)	0.0252 (15)	
H11	0.218763	0.341629	0.503728	0.030*	
C12	−0.1236 (7)	0.4176 (3)	0.3225 (4)	0.0221 (16)	
C13	−0.2555 (7)	0.4430 (3)	0.2936 (4)	0.0285 (17)	
H13	−0.316736	0.420800	0.261935	0.034*	
C14	−0.2982 (7)	0.4997 (3)	0.3102 (4)	0.0301 (17)	
H14	−0.388184	0.516220	0.290682	0.036*	
C15	−0.2077 (7)	0.5315 (3)	0.3553 (4)	0.0285 (17)	
H15	−0.235382	0.569845	0.368976	0.034*	
C16	−0.0758 (7)	0.5066 (3)	0.3805 (4)	0.0240 (15)	
H16	−0.011791	0.529120	0.410727	0.029*	
C17	0.0776 (6)	0.3673 (3)	0.2711 (3)	0.0202 (14)	
C18	0.3124 (7)	0.4105 (3)	0.2816 (4)	0.0229 (15)	
H18	0.390211	0.429587	0.308338	0.027*	
C19	0.3291 (7)	0.3989 (3)	0.2185 (4)	0.0254 (16)	
H19	0.417417	0.408224	0.201853	0.030*	
C20	0.2116 (8)	0.3731 (3)	0.1795 (4)	0.0291 (16)	
H20	0.217064	0.365863	0.134917	0.035*	
C21	0.0883 (7)	0.3580 (3)	0.2057 (3)	0.0228 (15)	
H21	0.008142	0.340852	0.178669	0.027*	
C22	0.1941 (7)	0.5283 (3)	0.3151 (4)	0.0237 (15)	
H22	0.114435	0.510501	0.288261	0.028*	
C23	0.2566 (7)	0.5790 (3)	0.2935 (4)	0.0267 (16)	
H23	0.220306	0.596237	0.252841	0.032*	
C24	0.3736 (8)	0.6044 (3)	0.3324 (4)	0.0275 (16)	
H24	0.422351	0.638299	0.318170	0.033*	
C25	0.4178 (6)	0.5793 (3)	0.3922 (3)	0.0193 (14)	
H25	0.496906	0.596920	0.419516	0.023*	
C26	0.3498 (6)	0.5285 (3)	0.4140 (3)	0.0170 (14)	
C27	0.2516 (6)	0.4826 (3)	0.5166 (3)	0.0164 (13)	
C28	0.2354 (7)	0.4966 (3)	0.5807 (3)	0.0220 (15)	
H28	0.312779	0.516530	0.606831	0.026*	
C29	0.1107 (7)	0.4822 (3)	0.6072 (4)	0.0285 (16)	
H29	0.101608	0.492171	0.650882	0.034*	
C30	−0.0007 (7)	0.4530 (3)	0.5686 (4)	0.0274 (16)	
H30	−0.088733	0.443125	0.585157	0.033*	
C31	0.0173 (7)	0.4388 (3)	0.5073 (4)	0.0224 (15)	
H31	−0.059511	0.418612	0.481070	0.027*	
C32	0.4590 (6)	0.4319 (3)	0.4654 (3)	0.0157 (14)	
C33	0.5985 (8)	0.4097 (4)	0.4851 (5)	0.052 (3)	
H33	0.665919	0.433211	0.512878	0.062*	
C34	0.6412 (9)	0.3542 (4)	0.4653 (6)	0.059 (3)	
H34	0.736993	0.339843	0.479238	0.070*	
C35	0.5451 (7)	0.3205 (3)	0.4258 (4)	0.0255 (16)	
H35	0.572332	0.282100	0.411915	0.031*	
C36	0.4093 (8)	0.3426 (3)	0.4065 (4)	0.0212 (15)	
H36	0.341834	0.319158	0.378721	0.025*	
B1	−0.0656 (7)	0.3510 (3)	0.3071 (4)	0.0194 (16)	
B2	0.3980 (7)	0.4969 (3)	0.4840 (4)	0.0159 (15)	
Co2_5	1.2099 (16)	0.8480 (7)	0.6903 (8)	0.039 (3)	0.369 (17)
O1_5	1.310 (2)	0.7702 (8)	0.5926 (9)	0.048 (5)	0.369 (17)
O2_5	0.9259 (17)	0.8949 (10)	0.6339 (15)	0.066 (6)	0.369 (17)
O3_5	1.206 (3)	0.7871 (19)	0.8129 (15)	0.047 (6)	0.369 (17)
O4_5	1.408 (2)	0.9501 (9)	0.7151 (18)	0.032 (5)	0.369 (17)
C46_5	1.270 (2)	0.8005 (10)	0.6312 (12)	0.038 (4)	0.369 (17)
C47_5	1.037 (2)	0.8750 (11)	0.6579 (16)	0.044 (4)	0.369 (17)
C48_5	1.206 (4)	0.8097 (16)	0.7619 (14)	0.040 (4)	0.369 (17)
C49_5	1.328 (3)	0.9097 (10)	0.7049 (19)	0.034 (4)	0.369 (17)
Co2_6	1.2050 (8)	0.8440 (4)	0.7021 (3)	0.0239 (10)	0.631 (17)
O1_6	1.2172 (18)	0.7556 (5)	0.6000 (5)	0.064 (4)	0.631 (17)
O2_6	0.9458 (10)	0.9188 (5)	0.6774 (6)	0.042 (3)	0.631 (17)
O3_6	1.2367 (19)	0.7831 (10)	0.8280 (7)	0.039 (3)	0.631 (17)
O4_6	1.4403 (13)	0.9316 (6)	0.7139 (9)	0.036 (3)	0.631 (17)
C46_6	1.2139 (17)	0.7907 (6)	0.6405 (6)	0.037 (3)	0.631 (17)
C47_6	1.0446 (11)	0.8877 (6)	0.6873 (7)	0.025 (2)	0.631 (17)
C48_6	1.219 (2)	0.8073 (9)	0.7772 (7)	0.028 (2)	0.631 (17)
C49_6	1.3487 (14)	0.8959 (6)	0.7087 (9)	0.026 (3)	0.631 (17)
Cl1_1	1.0632 (14)	0.6501 (5)	0.4497 (7)	0.110 (4)	0.451 (6)
Cl2_1	0.9607 (6)	0.7228 (3)	0.5467 (3)	0.066 (2)	0.451 (6)
C1_1	0.904 (3)	0.6809 (17)	0.4782 (14)	0.087 (6)	0.451 (6)
H1A_1	0.850180	0.706542	0.444319	0.104*	0.451 (6)
H1B_1	0.838297	0.648450	0.488647	0.104*	0.451 (6)
Cl1_2	1.0929 (8)	0.6542 (3)	0.4223 (4)	0.0492 (15)	0.549 (6)
Cl2_2	0.9031 (7)	0.6191 (2)	0.5147 (3)	0.0768 (19)	0.549 (6)
C1_2	0.958 (2)	0.6787 (9)	0.4713 (9)	0.046 (4)	0.549 (6)
H1A_2	0.999634	0.710368	0.501401	0.056*	0.549 (6)
H1B_2	0.872815	0.695525	0.443254	0.056*	0.549 (6)
C1_3	0.5022 (16)	0.5381 (8)	0.5352 (9)	0.016 (3)	0.549 (6)
C2_3	0.4597 (18)	0.5972 (8)	0.5414 (9)	0.018 (3)	0.549 (6)
H2_3	0.378877	0.611885	0.512764	0.022*	0.549 (6)
C3_3	0.530 (2)	0.6357 (11)	0.5876 (13)	0.021 (3)	0.549 (6)
H3_3	0.493817	0.675021	0.591754	0.025*	0.549 (6)
C4_3	0.6538 (13)	0.6172 (5)	0.6276 (6)	0.022 (2)	0.549 (6)
H4_3	0.709056	0.644470	0.655940	0.027*	0.549 (6)
C5_3	0.6943 (12)	0.5583 (5)	0.6252 (6)	0.019 (2)	0.549 (6)
H5_3	0.775145	0.544146	0.654155	0.023*	0.549 (6)
C6_3	0.6188 (11)	0.5192 (5)	0.5810 (6)	0.018 (2)	0.549 (6)
H6_3	0.646932	0.478370	0.581782	0.022*	0.549 (6)
C1_4	0.506 (2)	0.5441 (10)	0.5268 (13)	0.024 (3)	0.451 (6)
C2_4	0.446 (2)	0.5951 (10)	0.5513 (12)	0.024 (3)	0.451 (6)
H2_4	0.342908	0.600432	0.543357	0.029*	0.451 (6)
C3_4	0.529 (3)	0.6385 (13)	0.5870 (18)	0.027 (3)	0.451 (6)
H3_4	0.483178	0.673616	0.600335	0.033*	0.451 (6)
C4_4	0.6780 (18)	0.6309 (7)	0.6029 (9)	0.030 (3)	0.451 (6)
H4_4	0.733622	0.657263	0.632241	0.036*	0.451 (6)
C5_4	0.7426 (16)	0.5845 (6)	0.5756 (8)	0.031 (3)	0.451 (6)
H5_4	0.845933	0.580670	0.582671	0.037*	0.451 (6)
C6_4	0.6590 (15)	0.5418 (6)	0.5367 (8)	0.028 (3)	0.451 (6)
H6_4	0.707624	0.510871	0.516811	0.033*	0.451 (6)

### Bis[phenyltris(pyridin-2-yl)borato]cobalt(III) tetracarbonylcobalt(-I) dichloromethane monosolvate Atomic displacement parameters (Å^2^)

**Table:**

	*U*^11^	*U*^22^	*U*^33^	*U*^12^	*U*^13^	*U*^23^
Co1	0.0131 (4)	0.0176 (4)	0.0211 (5)	0.0003 (3)	−0.0035 (3)	−0.0019 (4)
N1	0.021 (3)	0.020 (3)	0.021 (3)	0.004 (2)	0.001 (2)	0.004 (2)
N2	0.016 (2)	0.017 (3)	0.023 (3)	0.001 (2)	−0.001 (2)	−0.002 (2)
N3	0.014 (2)	0.017 (3)	0.019 (3)	0.001 (2)	−0.004 (2)	0.000 (2)
N4	0.017 (3)	0.020 (3)	0.023 (3)	−0.001 (2)	−0.003 (2)	−0.005 (2)
N5	0.016 (2)	0.019 (3)	0.025 (3)	0.000 (2)	−0.001 (2)	0.000 (2)
N6	0.016 (3)	0.017 (3)	0.022 (3)	0.004 (2)	−0.003 (2)	0.000 (2)
C1	0.019 (3)	0.018 (3)	0.016 (4)	0.000 (2)	−0.002 (3)	0.004 (3)
C2	0.023 (3)	0.028 (4)	0.027 (4)	−0.004 (3)	0.003 (3)	−0.004 (3)
C3	0.021 (3)	0.035 (4)	0.037 (5)	−0.010 (3)	−0.002 (3)	−0.001 (4)
C4	0.031 (4)	0.028 (4)	0.036 (5)	−0.014 (3)	−0.006 (3)	−0.003 (3)
C5	0.033 (4)	0.018 (3)	0.025 (4)	−0.003 (3)	−0.003 (3)	0.000 (3)
C6	0.019 (3)	0.022 (3)	0.019 (4)	−0.001 (3)	0.001 (3)	0.002 (3)
C7	0.016 (3)	0.023 (3)	0.017 (4)	0.002 (3)	−0.002 (3)	−0.002 (3)
C8	0.029 (4)	0.025 (4)	0.033 (5)	−0.009 (3)	0.000 (3)	−0.004 (3)
C9	0.048 (5)	0.020 (4)	0.027 (5)	−0.013 (3)	−0.005 (4)	0.004 (3)
C10	0.041 (4)	0.030 (4)	0.026 (5)	−0.005 (3)	−0.009 (3)	0.008 (3)
C11	0.030 (4)	0.020 (3)	0.024 (4)	−0.002 (3)	−0.005 (3)	0.001 (3)
C12	0.018 (3)	0.021 (3)	0.026 (4)	−0.005 (3)	−0.001 (3)	−0.002 (3)
C13	0.019 (3)	0.023 (4)	0.041 (5)	0.001 (3)	−0.006 (3)	−0.007 (3)
C14	0.019 (3)	0.028 (4)	0.040 (5)	0.011 (3)	−0.007 (3)	0.002 (3)
C15	0.019 (3)	0.027 (4)	0.038 (5)	0.009 (3)	0.003 (3)	−0.004 (3)
C16	0.021 (3)	0.022 (4)	0.027 (4)	0.000 (3)	−0.006 (3)	0.000 (3)
C17	0.017 (3)	0.019 (3)	0.023 (4)	0.000 (2)	−0.004 (3)	0.004 (3)
C18	0.015 (3)	0.029 (4)	0.024 (4)	−0.002 (3)	−0.002 (3)	0.001 (3)
C19	0.018 (3)	0.036 (4)	0.022 (4)	−0.009 (3)	0.000 (3)	0.003 (3)
C20	0.030 (4)	0.041 (4)	0.015 (4)	−0.007 (3)	−0.003 (3)	0.005 (3)
C21	0.026 (3)	0.025 (3)	0.014 (4)	−0.008 (3)	−0.008 (3)	0.001 (3)
C22	0.020 (3)	0.023 (3)	0.026 (4)	0.006 (3)	−0.006 (3)	0.000 (3)
C23	0.032 (4)	0.022 (3)	0.023 (4)	0.003 (3)	−0.008 (3)	0.002 (3)
C24	0.029 (4)	0.022 (3)	0.031 (5)	−0.003 (3)	0.003 (3)	0.003 (3)
C25	0.017 (3)	0.017 (3)	0.023 (4)	−0.004 (2)	0.000 (3)	−0.006 (3)
C26	0.013 (3)	0.017 (3)	0.022 (4)	0.001 (2)	0.002 (3)	−0.002 (3)
C27	0.019 (3)	0.014 (3)	0.015 (4)	0.002 (2)	−0.003 (3)	−0.001 (3)
C28	0.017 (3)	0.029 (4)	0.020 (4)	−0.004 (3)	0.000 (3)	0.003 (3)
C29	0.022 (3)	0.044 (4)	0.020 (4)	−0.006 (3)	0.004 (3)	0.000 (3)
C30	0.019 (3)	0.036 (4)	0.027 (5)	−0.006 (3)	0.006 (3)	0.000 (3)
C31	0.015 (3)	0.025 (3)	0.026 (4)	−0.002 (3)	−0.001 (3)	−0.002 (3)
C32	0.011 (3)	0.021 (3)	0.015 (4)	−0.001 (2)	−0.001 (3)	0.004 (3)
C33	0.023 (4)	0.033 (4)	0.090 (8)	0.005 (3)	−0.028 (4)	−0.029 (5)
C34	0.022 (4)	0.037 (5)	0.109 (9)	0.008 (3)	−0.021 (5)	−0.028 (5)
C35	0.025 (3)	0.019 (3)	0.033 (4)	0.005 (3)	0.006 (3)	0.001 (3)
C36	0.025 (3)	0.018 (3)	0.020 (4)	0.001 (3)	0.001 (3)	−0.003 (3)
B1	0.013 (3)	0.018 (3)	0.027 (5)	−0.001 (3)	0.000 (3)	0.002 (3)
B2	0.014 (3)	0.017 (3)	0.016 (4)	−0.001 (3)	−0.003 (3)	−0.001 (3)
Co2_5	0.018 (3)	0.036 (3)	0.063 (6)	0.0030 (19)	0.008 (3)	0.009 (3)
O1_5	0.027 (8)	0.048 (9)	0.062 (10)	0.000 (7)	−0.014 (7)	−0.016 (7)
O2_5	0.025 (6)	0.068 (11)	0.104 (14)	0.006 (7)	0.008 (8)	0.051 (10)
O3_5	0.015 (10)	0.057 (11)	0.065 (12)	0.003 (9)	−0.008 (10)	0.018 (10)
O4_5	0.018 (8)	0.044 (9)	0.035 (9)	0.002 (6)	0.008 (8)	−0.007 (9)
C46_5	0.015 (6)	0.037 (6)	0.061 (8)	−0.002 (5)	0.003 (6)	0.004 (5)
C47_5	0.022 (5)	0.039 (7)	0.070 (9)	0.001 (5)	0.007 (6)	0.022 (6)
C48_5	0.018 (6)	0.039 (7)	0.064 (8)	0.003 (6)	0.006 (6)	0.011 (6)
C49_5	0.019 (6)	0.035 (6)	0.052 (8)	0.007 (5)	0.013 (6)	0.005 (6)
Co2_6	0.0159 (15)	0.0308 (17)	0.0237 (17)	−0.0011 (11)	−0.0021 (12)	0.0013 (13)
O1_6	0.090 (9)	0.057 (6)	0.041 (6)	0.011 (7)	−0.004 (6)	−0.015 (5)
O2_6	0.020 (4)	0.053 (6)	0.053 (7)	0.009 (4)	0.003 (5)	0.024 (5)
O3_6	0.026 (8)	0.056 (7)	0.031 (6)	0.005 (6)	−0.012 (5)	0.015 (5)
O4_6	0.023 (6)	0.056 (8)	0.028 (6)	−0.016 (5)	0.004 (5)	−0.001 (7)
C46_6	0.040 (5)	0.038 (5)	0.030 (5)	0.003 (4)	−0.006 (5)	−0.002 (4)
C47_6	0.016 (4)	0.034 (5)	0.024 (5)	−0.005 (3)	0.001 (4)	0.009 (4)
C48_6	0.019 (5)	0.035 (5)	0.027 (4)	−0.001 (4)	−0.006 (4)	0.005 (4)
C49_6	0.016 (4)	0.041 (5)	0.023 (5)	−0.002 (4)	0.005 (4)	−0.002 (5)
Cl1_1	0.085 (7)	0.058 (5)	0.180 (12)	0.005 (5)	−0.005 (7)	−0.050 (7)
Cl2_1	0.058 (3)	0.081 (4)	0.050 (4)	−0.028 (3)	−0.021 (3)	0.026 (3)
C1_1	0.074 (10)	0.067 (10)	0.113 (11)	−0.003 (9)	−0.012 (10)	−0.011 (10)
Cl1_2	0.055 (3)	0.024 (2)	0.074 (4)	0.0028 (19)	0.032 (3)	−0.003 (2)
Cl2_2	0.114 (4)	0.065 (3)	0.060 (3)	−0.024 (3)	0.040 (3)	0.001 (2)
C1_2	0.070 (8)	0.031 (6)	0.047 (7)	−0.004 (7)	0.040 (7)	0.000 (6)
C1_3	0.010 (4)	0.024 (5)	0.015 (5)	−0.002 (4)	0.004 (4)	0.001 (4)
C2_3	0.016 (5)	0.024 (5)	0.016 (5)	−0.001 (4)	0.003 (4)	0.004 (4)
C3_3	0.022 (5)	0.027 (5)	0.015 (5)	−0.003 (4)	0.005 (4)	0.001 (4)
C4_3	0.021 (4)	0.031 (4)	0.015 (5)	−0.009 (4)	0.002 (4)	0.000 (4)
C5_3	0.014 (4)	0.031 (4)	0.013 (5)	−0.004 (3)	0.000 (3)	−0.003 (4)
C6_3	0.014 (4)	0.027 (4)	0.014 (5)	0.000 (3)	0.003 (3)	−0.002 (4)
C1_4	0.024 (5)	0.018 (6)	0.029 (7)	−0.005 (5)	0.001 (5)	0.003 (5)
C2_4	0.026 (6)	0.018 (6)	0.029 (7)	−0.007 (5)	0.003 (6)	0.001 (6)
C3_4	0.030 (6)	0.020 (6)	0.030 (7)	−0.008 (5)	0.001 (6)	0.000 (5)
C4_4	0.031 (6)	0.023 (5)	0.033 (7)	−0.010 (5)	−0.006 (5)	0.001 (5)
C5_4	0.028 (5)	0.025 (5)	0.035 (6)	−0.004 (4)	−0.005 (5)	0.005 (5)
C6_4	0.025 (5)	0.024 (5)	0.033 (7)	−0.004 (4)	−0.002 (5)	0.004 (5)

### Bis[phenyltris(pyridin-2-yl)borato]cobalt(III) tetracarbonylcobalt(-I) dichloromethane monosolvate Geometric parameters (Å, º)

**Table:**

Co1—N1	1.937 (5)	C27—C28	1.397 (9)
Co1—N5	1.939 (6)	C27—B2	1.623 (9)
Co1—N4	1.960 (5)	C28—C29	1.379 (9)
Co1—N3	1.968 (6)	C28—H28	0.9500
Co1—N2	1.972 (5)	C29—C30	1.383 (10)
Co1—N6	1.979 (5)	C29—H29	0.9500
N1—C7	1.344 (8)	C30—C31	1.348 (10)
N1—C11	1.363 (9)	C30—H30	0.9500
N2—C12	1.348 (8)	C31—H31	0.9500
N2—C16	1.349 (8)	C32—C33	1.391 (9)
N3—C18	1.354 (8)	C32—B2	1.624 (9)
N3—C17	1.368 (8)	C33—C34	1.382 (11)
N4—C26	1.356 (8)	C33—H33	0.9500
N4—C22	1.356 (9)	C34—C35	1.357 (11)
N5—C27	1.351 (8)	C34—H34	0.9500
N5—C31	1.371 (8)	C35—C36	1.359 (9)
N6—C32	1.351 (8)	C35—H35	0.9500
N6—C36	1.353 (8)	C36—H36	0.9500
C1—C6	1.403 (9)	B2—C1_3	1.625 (18)
C1—C2	1.404 (9)	B2—C1_4	1.63 (2)
C1—B1	1.625 (9)	Co2_5—C48_5	1.725 (19)
C2—C3	1.387 (10)	Co2_5—C47_5	1.752 (17)
C2—H2	0.9500	Co2_5—C49_5	1.762 (17)
C3—C4	1.371 (10)	Co2_5—C46_5	1.769 (18)
C3—H3	0.9500	O1_5—C46_5	1.147 (19)
C4—C5	1.379 (10)	O2_5—C47_5	1.172 (19)
C4—H4	0.9500	O3_5—C48_5	1.18 (2)
C5—C6	1.382 (9)	O4_5—C49_5	1.169 (19)
C5—H5	0.9500	Co2_6—C49_6	1.755 (11)
C6—H6	0.9500	Co2_6—C48_6	1.757 (12)
C7—C8	1.411 (9)	Co2_6—C46_6	1.760 (12)
C7—B1	1.633 (10)	Co2_6—C47_6	1.767 (11)
C8—C9	1.383 (11)	O1_6—C46_6	1.156 (13)
C8—H8	0.9500	O2_6—C47_6	1.145 (12)
C9—C10	1.383 (10)	O3_6—C48_6	1.179 (14)
C9—H9	0.9500	O4_6—C49_6	1.157 (13)
C10—C11	1.375 (10)	Cl1_1—C1_1	1.79 (2)
C10—H10	0.9500	Cl2_1—C1_1	1.73 (3)
C11—H11	0.9500	C1_1—H1A_1	0.9900
C12—C13	1.405 (9)	C1_1—H1B_1	0.9900
C12—B1	1.629 (9)	Cl1_2—C1_2	1.797 (15)
C13—C14	1.386 (9)	Cl2_2—C1_2	1.724 (18)
C13—H13	0.9500	C1_2—H1A_2	0.9900
C14—C15	1.370 (10)	C1_2—H1B_2	0.9900
C14—H14	0.9500	C1_3—C2_3	1.391 (16)
C15—C16	1.378 (9)	C1_3—C6_3	1.406 (16)
C15—H15	0.9500	C2_3—C3_3	1.388 (16)
C16—H16	0.9500	C2_3—H2_3	0.9500
C17—C21	1.395 (10)	C3_3—C4_3	1.383 (18)
C17—B1	1.644 (9)	C3_3—H3_3	0.9500
C18—C19	1.368 (10)	C4_3—C5_3	1.371 (15)
C18—H18	0.9500	C4_3—H4_3	0.9500
C19—C20	1.392 (10)	C5_3—C6_3	1.389 (15)
C19—H19	0.9500	C5_3—H5_3	0.9500
C20—C21	1.368 (10)	C6_3—H6_3	0.9500
C20—H20	0.9500	C1_4—C2_4	1.394 (19)
C21—H21	0.9500	C1_4—C6_4	1.41 (2)
C22—C23	1.375 (9)	C2_4—C3_4	1.391 (18)
C22—H22	0.9500	C2_4—H2_4	0.9500
C23—C24	1.383 (10)	C3_4—C4_4	1.38 (2)
C23—H23	0.9500	C3_4—H3_4	0.9500
C24—C25	1.378 (10)	C4_4—C5_4	1.359 (18)
C24—H24	0.9500	C4_4—H4_4	0.9500
C25—C26	1.402 (8)	C5_4—C6_4	1.413 (18)
C25—H25	0.9500	C5_4—H5_4	0.9500
C26—B2	1.629 (10)	C6_4—H6_4	0.9500
			
N1—Co1—N5	89.0 (2)	N5—C27—B2	118.4 (6)
N1—Co1—N4	179.1 (2)	C28—C27—B2	123.8 (6)
N5—Co1—N4	91.1 (2)	C29—C28—C27	122.1 (6)
N1—Co1—N3	90.9 (2)	C29—C28—H28	119.0
N5—Co1—N3	179.9 (2)	C27—C28—H28	119.0
N4—Co1—N3	89.0 (2)	C28—C29—C30	118.3 (7)
N1—Co1—N2	91.3 (2)	C28—C29—H29	120.9
N5—Co1—N2	89.5 (2)	C30—C29—H29	120.9
N4—Co1—N2	89.6 (2)	C31—C30—C29	119.2 (6)
N3—Co1—N2	90.5 (2)	C31—C30—H30	120.4
N1—Co1—N6	88.7 (2)	C29—C30—H30	120.4
N5—Co1—N6	91.1 (2)	C30—C31—N5	122.5 (6)
N4—Co1—N6	90.4 (2)	C30—C31—H31	118.8
N3—Co1—N6	88.9 (2)	N5—C31—H31	118.8
N2—Co1—N6	179.4 (3)	N6—C32—C33	117.1 (6)
C7—N1—C11	120.4 (5)	N6—C32—B2	117.1 (5)
C7—N1—Co1	119.6 (5)	C33—C32—B2	125.8 (6)
C11—N1—Co1	119.9 (5)	C34—C33—C32	121.4 (7)
C12—N2—C16	120.4 (6)	C34—C33—H33	119.3
C12—N2—Co1	120.5 (4)	C32—C33—H33	119.3
C16—N2—Co1	119.0 (4)	C35—C34—C33	119.5 (7)
C18—N3—C17	120.0 (6)	C35—C34—H34	120.3
C18—N3—Co1	119.9 (4)	C33—C34—H34	120.3
C17—N3—Co1	119.7 (4)	C34—C35—C36	118.7 (6)
C26—N4—C22	120.2 (6)	C34—C35—H35	120.6
C26—N4—Co1	119.2 (4)	C36—C35—H35	120.6
C22—N4—Co1	120.3 (4)	N6—C36—C35	122.0 (6)
C27—N5—C31	120.3 (6)	N6—C36—H36	119.0
C27—N5—Co1	119.8 (4)	C35—C36—H36	119.0
C31—N5—Co1	119.9 (5)	C1—B1—C12	116.4 (5)
C32—N6—C36	121.3 (6)	C1—B1—C7	108.4 (5)
C32—N6—Co1	120.1 (4)	C12—B1—C7	107.2 (6)
C36—N6—Co1	118.6 (5)	C1—B1—C17	114.7 (6)
C6—C1—C2	114.4 (6)	C12—B1—C17	101.1 (5)
C6—C1—B1	122.7 (5)	C7—B1—C17	108.6 (5)
C2—C1—B1	122.3 (6)	C27—B2—C32	105.0 (5)
C3—C2—C1	122.9 (7)	C27—B2—C1_3	106.8 (8)
C3—C2—H2	118.5	C32—B2—C1_3	118.1 (7)
C1—C2—H2	118.5	C27—B2—C26	108.4 (5)
C4—C3—C2	120.3 (7)	C32—B2—C26	103.8 (5)
C4—C3—H3	119.8	C1_3—B2—C26	114.1 (8)
C2—C3—H3	119.8	C27—B2—C1_4	112.3 (11)
C3—C4—C5	118.9 (7)	C32—B2—C1_4	120.3 (9)
C3—C4—H4	120.5	C26—B2—C1_4	106.4 (10)
C5—C4—H4	120.5	C48_5—Co2_5—C47_5	112.4 (14)
C4—C5—C6	120.4 (7)	C48_5—Co2_5—C49_5	108.8 (16)
C4—C5—H5	119.8	C47_5—Co2_5—C49_5	108.0 (13)
C6—C5—H5	119.8	C48_5—Co2_5—C46_5	110.3 (14)
C5—C6—C1	122.9 (6)	C47_5—Co2_5—C46_5	107.0 (13)
C5—C6—H6	118.6	C49_5—Co2_5—C46_5	110.3 (14)
C1—C6—H6	118.6	O1_5—C46_5—Co2_5	179 (2)
N1—C7—C8	118.7 (6)	O2_5—C47_5—Co2_5	176 (2)
N1—C7—B1	118.8 (5)	O3_5—C48_5—Co2_5	175 (4)
C8—C7—B1	122.5 (6)	O4_5—C49_5—Co2_5	179 (3)
C9—C8—C7	120.7 (7)	C49_6—Co2_6—C48_6	105.8 (8)
C9—C8—H8	119.6	C49_6—Co2_6—C46_6	113.5 (8)
C7—C8—H8	119.6	C48_6—Co2_6—C46_6	109.1 (8)
C8—C9—C10	119.4 (6)	C49_6—Co2_6—C47_6	104.8 (7)
C8—C9—H9	120.3	C48_6—Co2_6—C47_6	112.0 (8)
C10—C9—H9	120.3	C46_6—Co2_6—C47_6	111.5 (7)
C11—C10—C9	118.2 (7)	O1_6—C46_6—Co2_6	178.9 (14)
C11—C10—H10	120.9	O2_6—C47_6—Co2_6	176.0 (11)
C9—C10—H10	120.9	O3_6—C48_6—Co2_6	176.4 (15)
N1—C11—C10	122.5 (7)	O4_6—C49_6—Co2_6	177.6 (14)
N1—C11—H11	118.7	Cl2_1—C1_1—Cl1_1	108.0 (14)
C10—C11—H11	118.7	Cl2_1—C1_1—H1A_1	110.1
N2—C12—C13	118.0 (6)	Cl1_1—C1_1—H1A_1	110.1
N2—C12—B1	116.8 (6)	Cl2_1—C1_1—H1B_1	110.1
C13—C12—B1	125.1 (6)	Cl1_1—C1_1—H1B_1	110.1
C14—C13—C12	121.5 (7)	H1A_1—C1_1—H1B_1	108.4
C14—C13—H13	119.2	Cl2_2—C1_2—Cl1_2	109.7 (10)
C12—C13—H13	119.2	Cl2_2—C1_2—H1A_2	109.7
C15—C14—C13	118.5 (6)	Cl1_2—C1_2—H1A_2	109.7
C15—C14—H14	120.7	Cl2_2—C1_2—H1B_2	109.7
C13—C14—H14	120.7	Cl1_2—C1_2—H1B_2	109.7
C14—C15—C16	118.6 (6)	H1A_2—C1_2—H1B_2	108.2
C14—C15—H15	120.7	C2_3—C1_3—C6_3	115.0 (14)
C16—C15—H15	120.7	C2_3—C1_3—B2	116.8 (12)
N2—C16—C15	122.7 (6)	C6_3—C1_3—B2	127.5 (13)
N2—C16—H16	118.6	C3_3—C2_3—C1_3	122.8 (15)
C15—C16—H16	118.6	C3_3—C2_3—H2_3	118.6
N3—C17—C21	117.9 (6)	C1_3—C2_3—H2_3	118.6
N3—C17—B1	116.7 (6)	C4_3—C3_3—C2_3	120.4 (15)
C21—C17—B1	125.2 (6)	C4_3—C3_3—H3_3	119.8
N3—C18—C19	123.2 (6)	C2_3—C3_3—H3_3	119.8
N3—C18—H18	118.4	C5_3—C4_3—C3_3	118.3 (13)
C19—C18—H18	118.4	C5_3—C4_3—H4_3	120.9
C18—C19—C20	117.4 (6)	C3_3—C4_3—H4_3	120.9
C18—C19—H19	121.3	C4_3—C5_3—C6_3	120.9 (11)
C20—C19—H19	121.3	C4_3—C5_3—H5_3	119.6
C21—C20—C19	119.7 (7)	C6_3—C5_3—H5_3	119.6
C21—C20—H20	120.1	C5_3—C6_3—C1_3	122.2 (12)
C19—C20—H20	120.1	C5_3—C6_3—H6_3	118.9
C20—C21—C17	121.6 (6)	C1_3—C6_3—H6_3	118.9
C20—C21—H21	119.2	C2_4—C1_4—C6_4	114.5 (18)
C17—C21—H21	119.2	C2_4—C1_4—B2	119.3 (16)
N4—C22—C23	122.7 (7)	C6_4—C1_4—B2	125.9 (18)
N4—C22—H22	118.7	C3_4—C2_4—C1_4	123.4 (19)
C23—C22—H22	118.7	C3_4—C2_4—H2_4	118.3
C22—C23—C24	118.4 (7)	C1_4—C2_4—H2_4	118.3
C22—C23—H23	120.8	C4_4—C3_4—C2_4	120 (2)
C24—C23—H23	120.8	C4_4—C3_4—H3_4	119.9
C25—C24—C23	118.5 (6)	C2_4—C3_4—H3_4	119.9
C25—C24—H24	120.7	C5_4—C4_4—C3_4	118.2 (16)
C23—C24—H24	120.7	C5_4—C4_4—H4_4	120.9
C24—C25—C26	122.0 (6)	C3_4—C4_4—H4_4	120.9
C24—C25—H25	119.0	C4_4—C5_4—C6_4	121.2 (14)
C26—C25—H25	119.0	C4_4—C5_4—H5_4	119.4
N4—C26—C25	117.9 (6)	C6_4—C5_4—H5_4	119.4
N4—C26—B2	118.0 (5)	C1_4—C6_4—C5_4	121.6 (16)
C25—C26—B2	124.1 (6)	C1_4—C6_4—H6_4	119.2
N5—C27—C28	117.7 (5)	C5_4—C6_4—H6_4	119.2
			
C6—C1—C2—C3	−3.6 (10)	C2—C1—B1—C12	−36.4 (9)
B1—C1—C2—C3	−175.2 (7)	C6—C1—B1—C7	−86.5 (7)
C1—C2—C3—C4	1.1 (12)	C2—C1—B1—C7	84.4 (7)
C2—C3—C4—C5	1.3 (12)	C6—C1—B1—C17	35.1 (9)
C3—C4—C5—C6	−1.0 (11)	C2—C1—B1—C17	−154.0 (6)
C4—C5—C6—C1	−1.8 (11)	N2—C12—B1—C1	171.1 (6)
C2—C1—C6—C5	3.9 (10)	C13—C12—B1—C1	−12.3 (10)
B1—C1—C6—C5	175.5 (6)	N2—C12—B1—C7	49.6 (7)
C11—N1—C7—C8	0.5 (9)	C13—C12—B1—C7	−133.8 (7)
Co1—N1—C7—C8	−175.6 (5)	N2—C12—B1—C17	−64.0 (8)
C11—N1—C7—B1	179.9 (6)	C13—C12—B1—C17	112.6 (7)
Co1—N1—C7—B1	3.8 (8)	N1—C7—B1—C1	176.6 (5)
N1—C7—C8—C9	0.4 (10)	C8—C7—B1—C1	−4.0 (8)
B1—C7—C8—C9	−179.0 (7)	N1—C7—B1—C12	−57.0 (7)
C7—C8—C9—C10	−1.2 (11)	C8—C7—B1—C12	122.4 (6)
C8—C9—C10—C11	1.1 (12)	N1—C7—B1—C17	51.4 (8)
C7—N1—C11—C10	−0.6 (10)	C8—C7—B1—C17	−129.2 (6)
Co1—N1—C11—C10	175.6 (6)	N3—C17—B1—C1	−169.7 (5)
C9—C10—C11—N1	−0.3 (11)	C21—C17—B1—C1	15.1 (9)
C16—N2—C12—C13	5.0 (10)	N3—C17—B1—C12	64.3 (7)
Co1—N2—C12—C13	−169.8 (5)	C21—C17—B1—C12	−110.9 (7)
C16—N2—C12—B1	−178.2 (6)	N3—C17—B1—C7	−48.2 (7)
Co1—N2—C12—B1	7.0 (8)	C21—C17—B1—C7	136.6 (6)
N2—C12—C13—C14	−4.6 (11)	N5—C27—B2—C32	−58.4 (7)
B1—C12—C13—C14	178.9 (7)	C28—C27—B2—C32	117.3 (6)
C12—C13—C14—C15	0.8 (12)	N5—C27—B2—C1_3	175.5 (8)
C13—C14—C15—C16	2.4 (11)	C28—C27—B2—C1_3	−8.9 (10)
C12—N2—C16—C15	−1.9 (10)	N5—C27—B2—C26	52.0 (7)
Co1—N2—C16—C15	173.0 (6)	C28—C27—B2—C26	−132.3 (6)
C14—C15—C16—N2	−2.0 (11)	N5—C27—B2—C1_4	169.2 (9)
C18—N3—C17—C21	−5.0 (9)	C28—C27—B2—C1_4	−15.1 (12)
Co1—N3—C17—C21	168.1 (5)	N6—C32—B2—C27	57.3 (7)
C18—N3—C17—B1	179.5 (5)	C33—C32—B2—C27	−124.6 (8)
Co1—N3—C17—B1	−7.5 (7)	N6—C32—B2—C1_3	176.1 (9)
C17—N3—C18—C19	1.5 (10)	C33—C32—B2—C1_3	−5.8 (13)
Co1—N3—C18—C19	−171.6 (5)	N6—C32—B2—C26	−56.4 (7)
N3—C18—C19—C20	2.4 (10)	C33—C32—B2—C26	121.7 (8)
C18—C19—C20—C21	−2.7 (11)	N6—C32—B2—C1_4	−175.1 (12)
C19—C20—C21—C17	−0.8 (11)	C33—C32—B2—C1_4	3.0 (16)
N3—C17—C21—C20	4.7 (10)	N4—C26—B2—C27	−48.5 (7)
B1—C17—C21—C20	179.8 (6)	C25—C26—B2—C27	134.6 (6)
C26—N4—C22—C23	3.4 (10)	N4—C26—B2—C32	62.7 (6)
Co1—N4—C22—C23	−171.2 (5)	C25—C26—B2—C32	−114.2 (6)
N4—C22—C23—C24	0.7 (10)	N4—C26—B2—C1_3	−167.5 (8)
C22—C23—C24—C25	−2.9 (10)	C25—C26—B2—C1_3	15.6 (10)
C23—C24—C25—C26	1.2 (10)	N4—C26—B2—C1_4	−169.5 (10)
C22—N4—C26—C25	−5.0 (9)	C25—C26—B2—C1_4	13.6 (12)
Co1—N4—C26—C25	169.6 (4)	C27—B2—C1_3—C2_3	−73.5 (17)
C22—N4—C26—B2	177.9 (5)	C32—B2—C1_3—C2_3	168.7 (13)
Co1—N4—C26—B2	−7.5 (7)	C26—B2—C1_3—C2_3	46.3 (18)
C24—C25—C26—N4	2.8 (9)	C27—B2—C1_3—C6_3	96.5 (16)
C24—C25—C26—B2	179.7 (6)	C32—B2—C1_3—C6_3	−21 (2)
C31—N5—C27—C28	3.5 (9)	C26—B2—C1_3—C6_3	−143.7 (15)
Co1—N5—C27—C28	−173.8 (4)	C6_3—C1_3—C2_3—C3_3	2.4 (18)
C31—N5—C27—B2	179.4 (5)	B2—C1_3—C2_3—C3_3	173.7 (17)
Co1—N5—C27—B2	2.2 (7)	C1_3—C2_3—C3_3—C4_3	4 (2)
N5—C27—C28—C29	−2.5 (9)	C2_3—C3_3—C4_3—C5_3	−7 (3)
B2—C27—C28—C29	−178.2 (6)	C3_3—C4_3—C5_3—C6_3	4 (2)
C27—C28—C29—C30	0.2 (10)	C4_3—C5_3—C6_3—C1_3	2.6 (19)
C28—C29—C30—C31	1.1 (11)	C2_3—C1_3—C6_3—C5_3	−6 (2)
C29—C30—C31—N5	−0.1 (11)	B2—C1_3—C6_3—C5_3	−175.8 (13)
C27—N5—C31—C30	−2.3 (10)	C27—B2—C1_4—C2_4	−45 (2)
Co1—N5—C31—C30	174.9 (5)	C32—B2—C1_4—C2_4	−169.3 (17)
C36—N6—C32—C33	0.4 (10)	C26—B2—C1_4—C2_4	73 (2)
Co1—N6—C32—C33	179.8 (6)	C27—B2—C1_4—C6_4	142 (2)
C36—N6—C32—B2	178.6 (6)	C32—B2—C1_4—C6_4	17 (3)
Co1—N6—C32—B2	−1.9 (8)	C26—B2—C1_4—C6_4	−100 (2)
N6—C32—C33—C34	−0.3 (14)	C6_4—C1_4—C2_4—C3_4	−4 (2)
B2—C32—C33—C34	−178.4 (9)	B2—C1_4—C2_4—C3_4	−178 (2)
C32—C33—C34—C35	−0.1 (16)	C1_4—C2_4—C3_4—C4_4	−4 (3)
C33—C34—C35—C36	0.4 (15)	C2_4—C3_4—C4_4—C5_4	9 (4)
C32—N6—C36—C35	−0.1 (10)	C3_4—C4_4—C5_4—C6_4	−6 (3)
Co1—N6—C36—C35	−179.6 (5)	C2_4—C1_4—C6_4—C5_4	8 (3)
C34—C35—C36—N6	−0.3 (12)	B2—C1_4—C6_4—C5_4	−179.0 (17)
C6—C1—B1—C12	152.7 (6)	C4_4—C5_4—C6_4—C1_4	−3 (3)
