# Supplementary material for: [Tpy2Co][Co(CO)4]: a mixed-valent cobalt(III)/cobalt(−I) com­plex based on phenyl­tris­(pyridin-2-yl)borate (Tpy−)
Source: Acta Crystallogr E Crystallogr Commun. 2026 May 22;82(Pt 6):712–6. doi: 10.1107/S2056989026004093 (PMC13238981; doi:10.1107/S2056989026004093)
Supplement: Supplementary file 3 [file e-82-00712-sup3.pdf]

[Tpy_2Co][$Co(CO)_4$]: A Mixed valent Co(III/–I) Complex Based on Phenyl *Tris*(2-pyridyl)borate (Tpy^-)

Oshani Wijesinghe[1], Robert J. Comito[1]*

Department of Chemistry, The University of Houston, Houston, Texas 77204

Supporting Information

Contents

1. Spectral data of [Tpy_2Co][$Co(CO)_4$] (**3**) S2

S1. Spectral Data for $[Tpy_2Co][Co(CO)_4]$ (3)

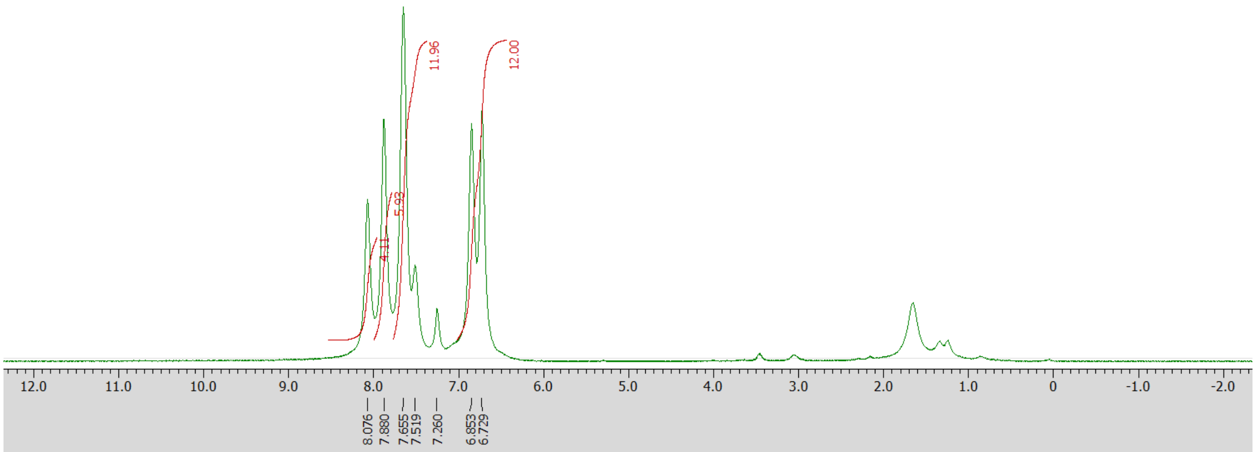

Figure S1. ^{1}H-NMR spectrum of $[Tpy_2Co][Co(CO)_4]$ (**3**), ($CDCl_3$, 400 MHz) (trace amount of water and grease present as the impurities in the 1-2 ppm region)

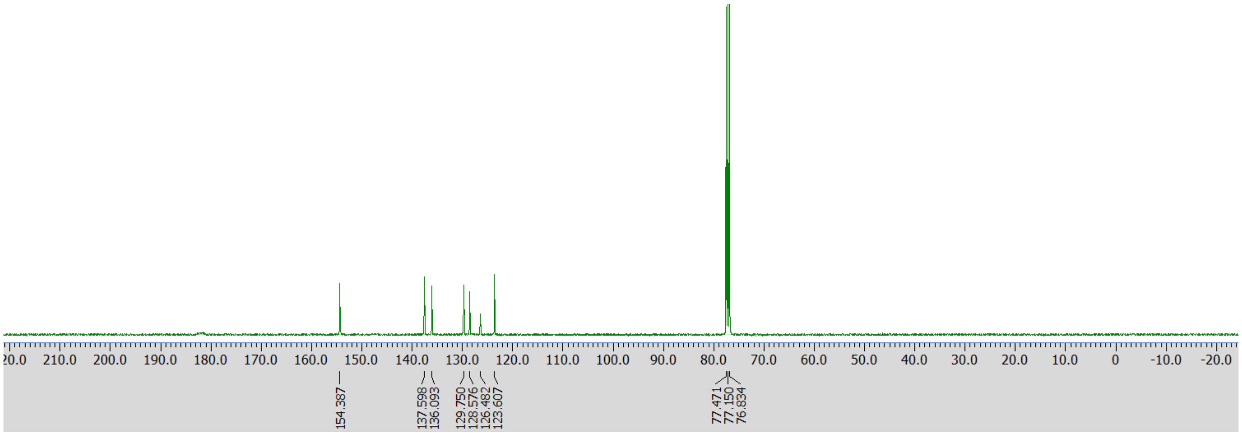

Figure S2. ^{13}C-NMR spectrum of $[Tpy_2Co][Co(CO)_4]$ (**3**), ($CDCl_3$, 101 MHz).

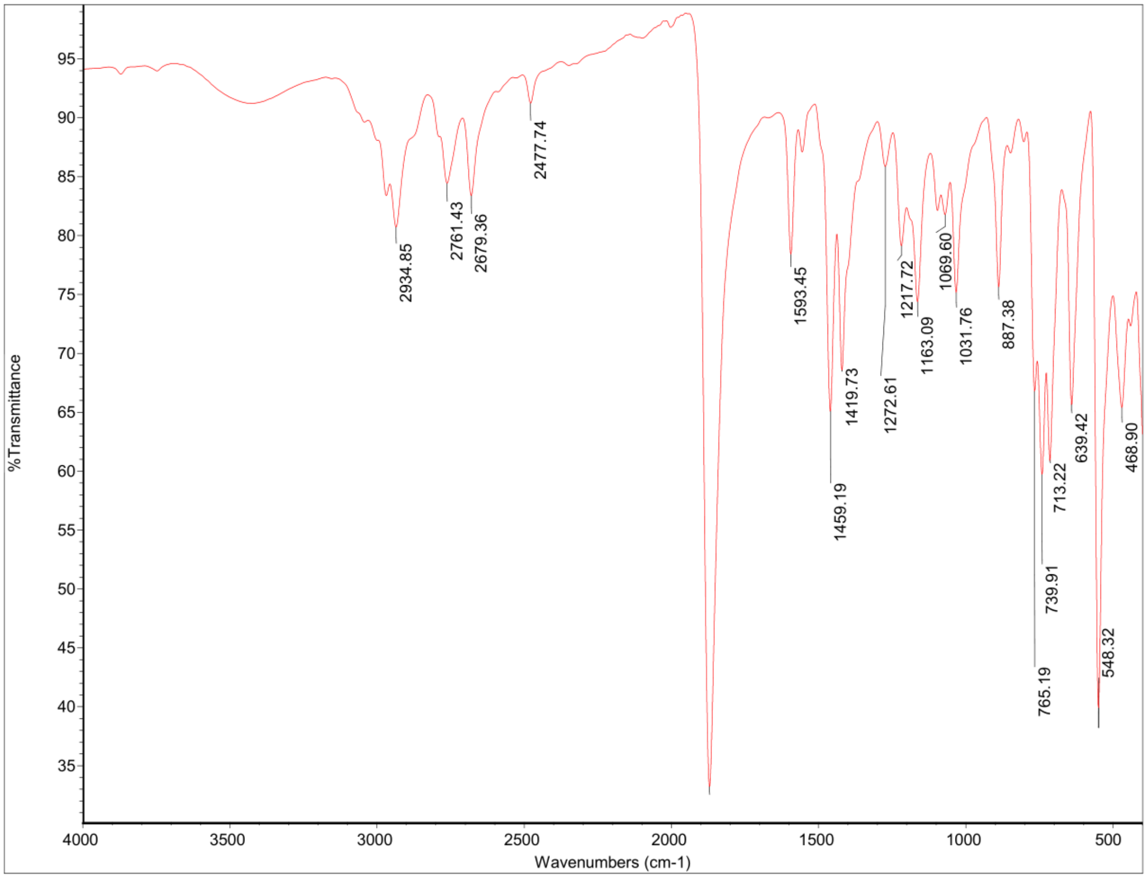

Figure S3. IR spectrum of [Tpy$_2$Co][Co(CO)$_4$] (**3**), diamond ATR.
